# Nutritional Status and Habits among People on Vegan, Lacto/Ovo-Vegetarian, Pescatarian and Traditional Diets

**DOI:** 10.3390/nu14214591

**Published:** 2022-11-01

**Authors:** Izabela Kwiatkowska, Jakub Olszak, Piotr Formanowicz, Dorota Formanowicz

**Affiliations:** 1 Department of Medical Chemistry and Laboratory Medicine, Poznan University of Medical Sciences, 61-701 Poznan, Poland; 2 Institute of Computing Science, Poznan University of Technology, 60-965 Poznan, Poland

**Keywords:** vegan diet, plant-based, vegetarian diet, lacto/ovo-vegetarian, body composition, diet quality, behavioral factors

## Abstract

**Background:** This study assessed the possible dependencies between nutritional habits and body composition among subjects with different dietary habits. **Materials:** A total of 196 healthy (aged 18–50 yrs) participants were enrolled in the study and divided into 4 groups according to their diet: vegans-VEGAN (*n* = 53), lacto/ovo-vegetarians—VEGE (*n* = 52), pescatarians-PESCA (*n* = 28), and omnivores-OMN (*n* = 43). **Methods:** The Food Frequency Questionnaire (FFQ) was used, and body composition was assessed on the In-Body120 analyzer. **Results:** Our result revealed in OMN + PESCA groups a higher average consumption frequency of sweets (*p* = 0.024), cheese/plant cheese (*p* < 0.001), eggs and egg dishes/egg substitutes (*p* < 0.001), butter, margarine/plant margarine (*p* < 0.001), cream /plant cream (*p* = 0.018), wine and cocktails (*p* = 0.028), vodka (*p* = 0.039) and lower of natural cottage cheese/tofu/tempeh (*p* < 0.001), vegetable oils (*p* = 0.036), legumes (*p* < 0.001) and nuts and seeds(*p* < 0.001) compared to the VEGAN + VEGE groups. The body composition analysis showed significant differences in skeletal muscle mass (SMM) (*p* = 0.019) and the content of minerals (*p* = 0.048) between groups. VEGAN disclosed the lowest average values of body fat mass (BFM), percentage body fat (PBF), and visceral adipose tissue (VAT) than other studied groups. **Conclusions:** The body composition analysis showed mean values within normal ranges in all of the groups, but some average results of OMN, PESCA, and VEGE compared to VEGAN were not highly satisfactory (in addition to eating behavior outcomes).

## 1. Introduction

Behavioral factors, which mainly include diet-related issues, tobacco, and alcohol use, and physical activity that can otherwise be defined as a lifestyle, demonstrate a significant impact on the possibility and frequency of disease and can determine life expectancy. According to the current data, compared to metabolic and environmental conditions, these factors significantly impact human health [1,2,3].

The WHO report [4] indicates health factors and behaviors (i.e., smoking, alcohol consumption, and excess body weight) as significant risk factors for premature deaths due to major non-communicable diseases, such as cardiovascular diseases, cancer, diabetes, and chronic respiratory diseases. This report shows that overweight and obesity in the European population are constantly increasing from 55.9% in 2010 to 58.7% in 2016 concerning overweight and from 20.8% to 23.3% in the same period for obesity [4].

The 2019 Global Burden of Disease Study (GBD) [5] has shown that behavioral factors are responsible for 49.35% of deaths from cardiovascular diseases, 45.29% of chronic respiratory diseases, and almost 1/3 of deaths from cancer (36.7%) and general causes (38.24%) worldwide, as estimated by using the Global Health Data Exchange (GHDx) [5,6].

In Poland, behavioral factors have an increased impact on the mortality rate compared to the world’s tendencies. The selected data are presented in Table 1.

For years, more and more new campaigns and programs regarding healthy behaviors in society have been created and implemented. They aim to indicate improvement strategies and increase social awareness of a healthy lifestyle. Over the past ten years, WHO has addressed recommendations on healthy eating, physical activity, and the adverse effects of tobacco and alcohol use [7,8,9,10,11,12]. However, although constantly updated and disseminated worldwide, these guidelines are still insufficient. The need for action to warn people against unhealthy diets, unsafe foods, and smoking must be emphasized in line with the urgent health challenges announced in 2020 for the next decade. It is also emphasized that, in addition to the problem of food shortage in one part of the world, excessive consumption of foods and drinks rich in sugar, saturated acids, trans fats, and salt is leading to an increasing trend of overweight and obesity worldwide. These aspects are responsible for 1/3 of the world’s diseases [13].

The problem of these mentioned extremes was also highlighted in The EAT-Lancet Commission Summary Report in 2019 [14], underlying that the negative health consequences of using a low-quality diet are the increased incidence of obesity and non-infectious diet-related diseases such as coronary heart disease, stroke, and diabetes. Food and its related aspects have been identified as one of today’s significant health and environmental challenges. Researchers [14] attribute a greater risk of morbidity and mortality instead to unhealthy diets than to the concomitant use of substances such as tobacco, alcohol, and drugs. The Global Panel on Agriculture and Food Systems for Nutrition report from 2016 [15] emphasizes the importance of nutritional systems/patterns among the population regarding their impact on poor health and environmental degradation. It highlights the need to transform the global food system. The objectives of another report [16] focus on food production’s ecological sustainability and the food’s health consequences eventually consumed. Among the five strategies presented, allowing for the described transformation, the first mentioned was the necessity to change the healthy diet on an international scale, taking into account the increased consumption of plant-based products and a significant reduction in the consumption of animal-derived food to achieve the establishments. It is necessary to invest in public health authorities, education about sustainable diet development, and collaboration between health and environmental departments [14,15,16].

The Lancet Countdown report from 2021 [17] on the red code for health and climate change also points to the need for changes to diet, further emphasizing the importance of the problem in highly developed countries, where the recommended change could bring tremendous benefits.

More and more reports [18,19,20,21,22,23,24,25] in the literature point to the possible beneficial effects of vegetarian diets on health, underlying their positive impact on prevention, health improvement, and the environment. Numerous research results indicate that people who follow vegetarian diets are characterized by a lower incidence ratio of cardiovascular diseases, cancer, and diabetes, as well as hypertension and obesity. An additional advantage of these diets may also be the fact that the results of studies on modifiable risk factors for cardiovascular diseases such as being overweight and obesity, parameters of the lipid profile-the concentration of total cholesterol, low-density lipoprotein cholesterol (LDL-C), and apolipoprotein B are lower in people on a vegetarian diet, primarily vegan, compared to traditional diets. Studies conducted by many researchers also show the positive effects of using plant-based diets as a therapeutic means [24,26,27,28,29,30,31,32,33,34,35].

The definition of a vegetarian diet indicates eliminating meat foods but allowing the consumption of eggs and dairy products. All varieties of these diets are characterized by higher consumption of plant products (whole grains, vegetables, fruits, legumes, nuts, and seeds) containing large amounts of fiber, vitamins, minerals, and phytochemical substances. In contrast, the products that are the source of saturated fatty acids are reduced or eliminated. There are numerous varieties of such a diet; the criterion for distinguishing them is the inclusion and elimination of a range of groups of food products in the daily diet. It is worth noting that if diets are adequately balanced, they can be used at any stage of life. Still, this position does not apply to extreme varieties (e.g., raw food, fruitarianism), which can be particularly dangerous to health and do not have scientific confirmation of possible health effects [19,36,37]. The following types of vegetarianism have been determined in the literature: semi-vegetarianism, pescetarianism, lacto-ovo-vegetarianism, lacto-vegetarianism, ovo-vegetarianism, veganism, and restrictive varieties (i.e., raw veganism, fruitarianism), which are characterized in Table 2.

While there are promising reports on vegetarian diets, they are still the subject of numerous extreme opinions since, despite the proven benefits of their use in many ways, not every vegetarian/vegan follows the recommendations. This applies primarily to the appropriately balanced intake of products; according to the views of world societies, only a properly balanced diet can bring health benefits. Giving up some food groups creates the risk of insufficient coverage of the demand for all nutrients. When nutritional deficiencies occur in an unbalanced vegetarian diet, the health benefits are nullified [19,38].

In the available literature from the last decade, there are reports [24,26,27,29,30,32,33,34] assessing the quality indicators of various vegetarian diets. However, very rarely do studies include different types of vegetarianism, including veganism. The most frequently quoted comparisons include vegetarians and non-vegetarians or vegans and non-vegans; only one available study [34] compared the varieties of diets, including semi-vegetarians, pescatarians, vegetarians, and vegans. The most frequently used methods of assessing diet quality in the cited studies are The Food Frequency Questionnaire (FFQ), Healthy Eating Index 2010 (HEI-2010), and the Mediterranean Diet Score (MDS). In line with the above, one of the valuable tools for assessing the individual usual dietary intake, commonly used in nutritional epidemiological studies, is the FFQ [39]. The FFQ is an advanced form of a diet history checklist that asks respondents how often and how much food they ate over a certain period. It enables researchers to categorize subjects according to food intake and the nutritional value of the products. Since the 1990s, various FFQs, as practical instruments, have been widely used since this method allows the assessment of long-term food consumption in a relatively simple, economical, and time-efficient way. In the meta-analysis, Cui et al. [40] assessed FFQs reproducibility among 20,542 participants from 123 studies. They revealed that FFQ measurement for most nutrients might be reliable for measuring dietary intake. This tool allowed for identifying nutritional patterns to find a relationship with health outcomes, see [40,41,42,43,44]. In the cited studies, the diet quality was analyzed without reference to the body composition. In only one study by Morgan-Bathke et al. [45], among eight obese vegetarians and eight obese omnivores, the assessment of diet quality, body composition, and adipose tissue inflammation was performed. The obtained results indicated that dietary patterns play a role in adipose tissue inflammation as reflected by the reduced number of femoral adipose macrophages in the vegetarian group (vegetarians consumed 42% less saturated fat and 50% less cholesterol than the omnivores (*p* = 0.02 and *p* = 0.04, respectively).

There are also more and more studies comparing body composition [46,47,48,49,50,51,52,53,54,55,56] in people with different nutritional regimens. The analyses in these studies are usually based on the respondents’ self-declaration. The most recent meta-analysis from 2022 conducted by Fontes et al. [47] has taken together studies assessing body composition in different dietary groups. The overall sample of participants consisted of 436,178 people, of whom 10,090 were vegetarians, and 5044 were vegans. Most studies have found a significant positive relationship between plant-based diets and body composition. It should be noted here that the meta-analysis has shown that two or three groups of participants with different nutrition preferences were usually compared in one study [48,49,50,51,55,56]. Moreover, most of these studies did not compare eating habits (only a few used FFQ). In these studies where such a comparison was made, it only included two groups: vegans and non-vegans [48,56]. The analysis of body composition in the available studies mainly includes the basic components of BMI, body weight, and waist circumference [48,49,50,51,52,53,56]. Only two studies considered muscle mass and lean body mass [54,55], and only one study [55] included bone mineral density in addition to the above-mentioned components. Hence, more extended analyzes are valuable.

The study aimed to assess and compare the frequency of consumption of selected food products among healthy young adults belonging to one of the selected groups: lacto/ovo-vegetarian, vegans, pescatarians, or omnivores, along with the in-depth analysis of body composition based on numerous parameters.

## 2. Materials and Methods

### 2.1. Study Design

The study was conducted in Poland during the COVID-19 pandemic period at the Poznan University of Medical Sciences (Department of Medical Chemistry and Laboratory Medicine) at the turn of June and July (24 June, 26 June, 29 June, and 13 July in 2021). This study investigated the possible dependencies between nutritional habits and body composition among subjects with different dietary habits.

The study was conducted in accordance with the Declaration of Helsinki, and the Bioethics Committee approved the study protocol of the Medical University (No. 237/20/2020).

Each adult individual encountered the study invitation via social media, and each participant self-identified their current diet by choosing from one of the available options: omnivore, vegetarian (‘lacto’ (e.g., included dairy products), ‘ovo’ (e.g., included eggs), ‘lacto-ovo’ (e.g., included dairy and eggs) ‘pesco’ (e.g., included fish and seafood), or vegan (not including dairy or eggs or fish and seafood). In this study, we included self-selected ‘healthy’ participants who voluntarily and anonymously decided to participate in the study according to the inclusion/exclusion criteria. Then participants came to the appointed day of the examination, where the body composition analysis was performed by a qualified nutritionist after reading the accompanying text and signing an informed consent form for inclusion in the study. After that, each participant was asked to complete the questionnaires. It should be pointed out that in the initial phase of the study, a comparison of the participants’ food diaries was planned, but with the pandemic leading to the limited availability of products, as well as a reluctance to fill in such a form or not fill in the diary meticulously due to stress, the idea was withdrawn. The primary tool used to evaluate the diet was the validated FFQ questionnaire. The participant completed the FFQ and Diet and behavioral factors questionnaire (Supplementary Materials, Questionnaires S1 and S2) that assessed the frequency of consumption of selected products and behavioral factors such as sleep duration, level of activity, or tobacco use. After the body composition analyses results, it turned out that not all participants were aware that obesity is also a disease, and it was necessary to exclude some people from the study since one of the study’s objectives was to compare mostly body weight, body fat content and other selected parameters among healthy participants. To support the obtained results, the triangulation of data was made (the Delphi methodology [57]); see Supplementary Materials (Questionnaire S3). Detailed data on the questionnaire responses in the analyzed groups have been included in Supplementary Materials (Table S1).

### 2.2. Subjects

In the online recruitment stage, 231 participants were enrolled. Still, after reading the applicable participation criteria, 35 people had to resign (due to age restrictions or chronic diseases in the interview).

The inclusion criteria for qualifying for the study were as follows: adults between 18 and 50 years (due to the burden of age-related diseases), informed consent to participate in the study, healthy (no chronic diseases diagnosed), participants should have had the same (current) dietary pattern for ≥ 1 year. The acute processes were also excluding factors. Pregnant and lactating women also did not participate in this study.

Finally, one hundred ninety-six participants were recruited for this study between April and July 2021. The recruited participants were divided into four groups depending on their diet: (1) mixed diet/omnivores [OMN] (traditional, including meat consumption), (2) pescatarian diet [PESCA] (including the consumption of fish, eggs, and dairy products among animal products), (3) lacto/ovo-vegetarian diet [VEGE] (eggs and dairy products including from animal products, this group includes: lacto-vegetarian, ovo-vegetarian or lacto-ovo-vegetarian), and (4) participants on a vegan diet/[vegan/-s, VEGAN] (not including meat products or animal origin). The type of diet was determined by the participants in the proprietary “Diet and Behavioral Factors Questionnaire,” through a personal declaration, to which type of diet is declared from possible choices (omnivore, vegetarian (‘lacto’,‘ovo’,’lacto-ovo’) pescatarian or vegan) and then compliance with the FFQ questionnaire was checked through the declared answers about the frequency of consumption, e.g., vegans chose the “never” answer with animal products, pescatarian declare to eat fish etc.)

**Figure 1:**
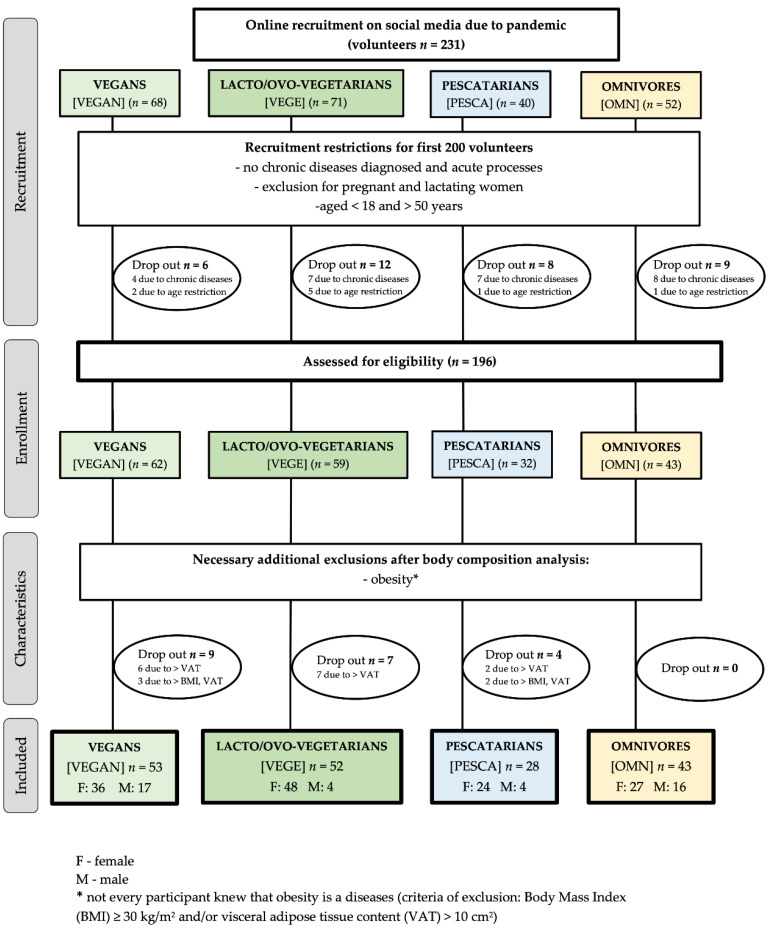
The scheme of recruiting participants.

Finally, 20 participants were excluded (9 vegans, 7 lacto/ovo-vegetarians, 4 pescatarians) due to incorrect body composition, indicating obesity. Ultimately, the number of respondents was 176. The recruitment scheme of the respondents is presented in Figure 1.

### 2.3. Body Composition Analysis

The participants underwent a body composition analysis using the InBody 120 analyzer (Seoul, Korea). The device uses the bioelectric impedance method (Direct Segmental Multi-frequency Bioelectrical Impedance Analysis Method, DSM-BIA method), thanks to which it was possible to evaluate parameters such as body weight [kg], muscle mass [kg], amount of adipose tissue [kg, %] including visceral adipose tissue [cm^2^].

Before starting the analysis, it is recommended to be fasting for 8–12 h, avoid fluid consumption for 1 h before the test, and refrain from excessive physical activity for 24 h. Contraindications for the analysis are implanted metal devices imitating the electromagnetic field (e.g., a pacemaker) or pregnancy. The participants were provided with this information before the test was performed, and no one reported any contraindications.

### 2.4. Diet and Behavioral Factors Questionnaire

During the study, participants were asked questions included in a short diet questionnaire regarding the declaration of the diet used (omnivore, pescatarian, lacto/ovo-vegetarian, or vegan), as well as behavioral factors, such as, e.g., physical activity, tobacco and alcohol use, or duration of sleep.

### 2.5. Dietary Intake-Assessment of Frequency of Consumption of Food Products Based on Food Frequency Questionnaire (FFQ)

To assess dietary habits in the past year, the Food Frequency Questionnaire (FFQ) was used, which was based on a validated 62-item FFQ [40].

Based on which particular questions (*n* = 37) from various food groups were selected regarding the consumption of certain products. Possible modifications were taken into account, e.g., adding plant substitutes for animal products to check the frequency of consumption in each study group (e.g., tofu, tempeh, vegetable yoghurt, egg substitutes), which was necessary due to the avoidance of traditional representatives of a given food group by some of the studied groups. The modifications applied were authorized by a qualified nutritionist. Possible responses ranged from “never/almost never” to “daily.” The mean frequency of consumption for each product in the study groups was calculated by assigning each possible answer a number from one to five (see Table S1).

### 2.6. Statistical Analysis

Statistical analysis was performed using the Jamovi statistical software [58]. The normal distribution in the Shapiro-Wilk test was violated for almost all variables. Additionally, Levene’s homogeneity test confirmed the violation of the assumption of equal variances. Due to these results, non-parametric methods were used in all analyzes. The Kruskal-Wallis One-Way ANOVA test was used to compare the results between the groups. Differences between each pair of groups were tested using the Dwass-Steel-Critchlow-Fligner (DSCF) post hoc test. Correlations between food consumption and body composition were calculated using the Spearman method. The Mann-Whitney U test was used to compare the body composition in each group depending on the high or low frequency of consumption of the tested products. The statistically significant results were set at *p* < 0.05. Additionally, using the statsmodel Python module [59], linear regression models and generalized linear models were calculated. The influence of selected groups of products (e.g., sweets, white bread, juices, alcohol) on a body composition chosen parameter (such as visceral) was tested. However, the effect of the selected products was often negligible and not statistically significant.

## 3. Results

### 3.1. Baseline Characteristics

Among 176 study participants, 53 participants (30.1%) adhered to a VEGAN, 52 (29.5%) of them were VEGE, 28 (15.9%) were PESCA and 43 (24.4%) were OMN. Table 3 shows the baseline characteristics of the participants following four different diets.

The results in the presented table were analyzed, but no statistical significance was demonstrated. The average age of the respondents was similar; hence no statistical differences in the respondents’ age were found, proving reliable sample analyzes (no impact of age). However, the division of the study participants into age categories was primarily planned; it had to be abandoned. The similar age in groups may be due to the place of recruitment-social media, which young adults or adults mainly use. The most significant number of respondents was in the age group 21–23 and 27–29.

Moreover, when gender was taken into account to check the differences between the groups, there were no statistically significant differences (not shown in the table).

The groups showed similar physical activity results, declaring the highest percentage of average physical activity. The VEGAN group was the least active, contrary to the OMN group, which was found to be the most active when comparing the results.

Most participants declared that they do not smoke cigarettes. Most often, the PESCA group used this type of substance. Almost 20% of this group reported regularly smoking small amounts (less than a pack/day).

Compared to other groups, VEGAN least often pointed the answer indicating too short sleep (below 6 h/per night), and more often than other respondents, they stated the daily sleep duration below nine hours per night. Besides, concerning the frequency of vodka consumption, in this group, the highest percentage (3/4 of respondents) indicated complete alcohol withdrawal, and the frequency of consumption of this product was the lowest in this group.

The average duration of adherence to the current diet among the study groups (participants on an OMN diet were omitted) was 3.9 years for VEGAN, 8.2 years for VEGE, and 5.6 years for PESCA. In addition, many vegans reported earlier adherence to a lacto/ovo-vegetarian diet; therefore, the duration of meat withdrawal in this group would eventually be longer/could be longer).

### 3.2. Body Composition Analysis Results

The parameters showing the nutritional status of the subjects were measured using the InBody body composition analyzer. The results for each group are presented in Table 4.

Next, the revealed BMI values among the study participants were divided into the categories of underweight, normal body weight, and overweight; the results are presented in Table 5.

The body composition results of all studied groups (taking into account obtained medium values) were within the normal ranges. The highest average values concerning adipose tissue content [kg], body weight, and BMI were revealed in the OMN group; the highest percentage of overweight and the lowest of underweight subjects were also observed in this group. The VEGAN group had the lowest adipose tissue content [kg], while the group of VEGE showed the lowest body weight and BMI value. The body fat percentage was the highest in the group of VEGE and the lowest in the VEGAN.

The respondents’ muscle content [kg] was the lowest in the VEGE group, while the highest muscle content was shown in an OMN and VEGAN group.

The WHR value and the degree of obesity were the highest in the OMN and PESCA groups. Besides, visceral fat was the highest in the PESCA group and the lowest among the members of the VEGAN group.

### 3.3. The Results of the Frequency of Consumption of Selected Products in the Studied Groups

**Scheme 1:**
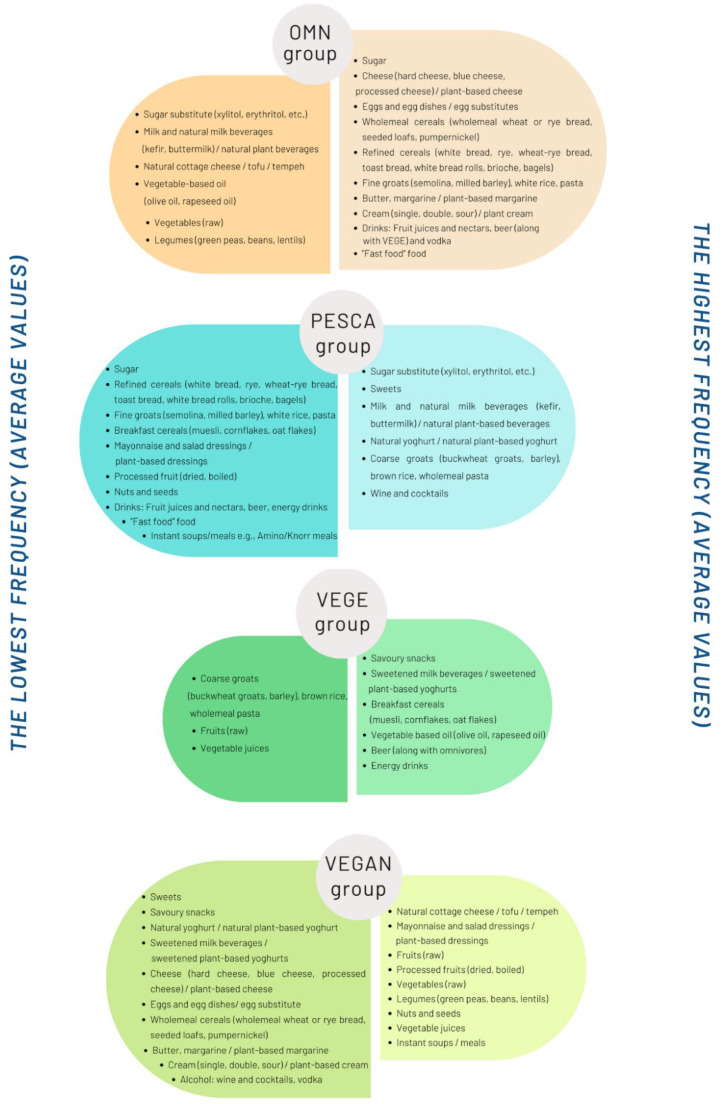
The highest and the lowest frequency of consumption between groups (results from the FFQ questionnaire).

The diet of the study groups was assessed based on the responses from the modified Food Frequency Questionnaire (FFQ) form. The cumulative mean results about the highest and the lowest frequency of consumed groups of products in the studied groups are shown in Scheme 1. The obtained results were subjected to statistical analysis to compare the significance of differences in the studied nutritional regimens.

The OMN group, more often than the other groups, used products such as sugar, cheese/plant-based cheeses, bread, processed cereal products, butter/margarine/plant-based margarine, cream/plant-based cream, fruit juices and nectars, vodka, and fast food. On the other hand, the consumption of milk and natural dairy products/plant-based drinks, cottage cheese/tofu/tempeh, vegetable oils, and vegetables, including legume seeds, was the least frequent in relation to other groups.

The VEGAN group, more often than other respondents, chose “natural cottage cheese/tofu/tempeh” which was a protein source category product, fruit (both raw and processed), vegetables, legume seeds, nuts, and grains. Although least frequently compared to the other groups, they reach for sweets, salty snacks, high-fat dairy products (cheese, butter, cream)/plant-based substitutes and eggs/eggs substitutes, wholemeal cereals, and alcoholic beverages such as wine, drinks, and vodka.

The results showing statistically significant differences in the frequency of consumption between the different groups are shown in Table 6.

Most of the differences in the frequency of consumption of the tested products (in each category, including sources for both traditional and vegetarian diets) were found between the OMN group and the VEGAN group. Significantly more often among the OMN group were consumed products that could be considered as generally assigned to this group, such as cheese/plant-based cheese, eggs, egg dishes/egg substitutes, butter, margarine/plant-based margarine, cream (single, double, sour)/plant-based cream but also refined cereals and wine and cocktails. The VEGAN group, on the other hand, significantly more often consumed (apart from products generally assigned to this group: legumes, nuts and seeds) natural cottage cheese/tofu/tempeh, compared to the OMN group.

Numerous significant differences were also found between the groups of VEGE and VEGAN. VEGE versus VEGAN differed in increased consumption of products which could be considered as generally assigned to this group, such as the following: cheese/plant-based cheese, eggs, and egg dishes/egg substitutes, butter, and margarine/plant-based margarine. However, the VEGE group showed significantly less consumption of fruits, legumes, and also nuts and seeds (which is also generally taken into account in a lacto/ovo-vegetarian diet).

Moreover, the studied groups were divided into two categories: the OMN + PESCA group as the group taking into account the consumption of meat and the VEGE + VEGAN group as the group not taking into account the consumption of animal products. The results are presented in Table 7.

The obtained results, showing the significance of the differences, confirmed the previously observed (based on average values) dependencies concerning the increased consumption in groups, including the consumption of meat, products such as sweets, cheese, butter, cream, and alcoholic beverages) than in the other groups; moreover, they showed increased consumption of eggs. The group VEGE + VEGAN revealed significantly more frequent consumption of products, such as natural cottage cheese/tofu/tempeh, vegetable oils, legumes, nuts and seeds, if compared to OMN + PESCA.

### 3.4. The Results Showing Relationships between the Parameters of the Body Composition and the Frequency of Selected Products Consumption in the Studied Groups—Data Showing Statistically Significant Correlations

The OMN group disclosed an inverse correlation between sugar consumption and body composition parameters such as minerals, SMM, and the consumption of healthy products from the HDG products category and the value of the BMI. This group also revealed significant positive correlations between the consumption of unhealthy drinks and the BMI and VAT index values. PESCA showed a positive correlation between the consumption of eggs and egg dishes/egg substitutes and SMM.

The VEGE group revealed a significant weekly positive correlation between sugar consumption and BMI and WHR as well as between high sugar products and BMI.

Among the VEGAN group, significant weak negative correlations were found between the consumption of sweets and instant soups/meals and the content SMM, and minerals. The described correlations are presented in Table 8.

In order to compare in detail the influence of food choices on the parameters of body composition analysis, all studied groups were divided into people representing different levels of consumption frequency—high (H) and low (L) in relation to selected food products. Group H includes the answers: “daily”, “several times a week,” and “several times a month”, while L group includes the answers: “once a month or less” and “never or almost never”. The results are presented in Table 9.

Additional relationships emerged when the described division in the OMN group was taken into account, including the following: the WHR was significantly lower in people who consumed whole grain cereal products more frequently, such as whole meal bread, compared to those whose consumption of these products was low; the consumption of nuts and grains in group H decreased the BMI and WHR index values and increased the percent of body fat compared to group L. The level of WHR in group H was dramatically raised by beer drinking. Consuming more milk and coarse groats was revealed to relate to body weight reduction.

The results observed in the PESCA group show that increased sugar consumption (group H) resulted in higher body weight and WHR value. In contrast, increased consumption of sugar substitutes (group H) resulted in lower body fat, including visceral fat. The greater fish consumption (group H) showed a higher content of minerals.

VEGE in group H has a higher body weight in relation to sugar consumption. Dairy products such as cottage cheese/tofu/tempeh in the H group increased muscle. Sweet cheese, if eaten at a lower frequency (group L) has been proven to lower values of BMI% body tissue and the degree of obesity in the subjects. On the other hand, reduced consumption of wholemeal cereal (group L) shows increased values of the BMI index and the degree of obesity.

In the VEGAN group, increased adipose tissue was observed for group H in relation to the consumption of milk and processed fruit, and for group L, regarding the consumption of breakfast cereals, nuts, and seeds. Consumption of sweets and ready-made meals in group H influenced the subjects’ lower minerals and muscles values.

Regarding wine consumption, in the PESCA and VEGAN groups, the muscle content was lower in the case of increased product consumption (group H). Minerals content showed opposite relationships between these groups; in the PESCA group, it was higher in the case of increased wine consumption (group H), and in the VEGAN group, in the case of reduced consumption (group L).

## 4. Discussion

The study’s results demonstrate differences between behavioral factors among the various diet groups and discovered associations in relation to the consumption of certain food groups and body composition parameters.

### 4.1. Behavioral Factors in the Study Groups

Behavioral factors (i.e., sleep duration, physical activity, and cigarette smoking) did not differ significantly among the subjects. On average, the least active group was VEGAN, the most active was the OMN group; cigarette smoking was higher among pescatarians relative to the other groups. Alcohol consumption, on the other hand, was the highest in the OMN group and the lowest in the VEGAN group. In the Adventist Health Study 2 (AHS-2) [22], it was observed that the lowest alcohol consumption and rarest cigarette smoking occurred among vegans (*n* = 5548) and vegetarians (*n* = 21,177) relative to the other groups studied (pescatarians, semi-vegetarians, and omnivores). In contrast, in terms of physical activity, the omnivore group (*n* = 35,359) was the least active, and no significant differences in sleep among the studied groups were observed. On the other hand, in the Xie et al. study [62], alcohol consumption was, much as in the conducted research, highest in the omnivore group, while cigarette smoking was highest among vegans; in terms of physical activity level, the mixed group was the least active. The above correlations may be due to more significant concern for health among VEGANs. As a result of higher health consciousness, these individuals seem to lead healthier lifestyles [63,64].

### 4.2. Comparison of Body Mass and Adipose Tissue (BFM, PBF), including Visceral Adipose Tissue (VAT)

People entering this study had to meet the inclusion criterion, considering the lack of chronic diseases, including obesity; hence a few pescatarians, lacto/ovo-vegetarian, and vegans were excluded from the study. The exclusion was necessary to compare body composition in the most meaningful trial possible.

The purpose of the comparisons is to identify possible trends; the study group is healthy and mainly young adults, which should be considered. Comparing the potential risk of obesity among the study groups, the OMN group had the highest average body weight, BMI, and WHR values. However, the results of these components (medium values) were within the normal range. The highest BMI value relative to the other groups was also observed among the omnivore group in the study: AHS-2 [22] (pescatarians, semi-vegetarians, lacto-ovo and vegan were additionally included), the survey by Rosi et al. [24] (lacto-ovo and vegan were additionally included) and the study by Dorard and Mathieu [64] (vegetarians and omnivores were included) or Bradbury et al. [18] while the higher BMI and WHR in the mixed group relative to the vegetarian groups (including vegan) was shown by Xie et al. [62]. In most of the available studies [47,49,50,53,55] comparing body composition parameters, non-vegetarian versus vegetarian groups showed higher BMI values in non-vegetarian groups.

In the Clarys et al. [34] study, vegans (*n* = 104) and vegetarians (*n* = 573), and in Dorard and Mathieu [64] study, vegetarians (*n* = 49) had the lowest percentage of being overweight, and the highest of underweight among the groups studied, as also shown in the results of the current study. Among VEGANs, the average weight percentage was very similar to the results of the Clarys et al. [34] study (i.e., 78.8% vs. 77.36% of the studied VEGANs participants). It is worth noting that in the cited study, VEGANs had the highest ratio of normal BMI scores among all groups. In the present study, the VEGE group revealed the highest percentage of the normal BMI score (88.46% of the VEGE participants. Still, it is also worth mentioning that the lowest average body weight is associated with this group’s lowest average muscle mass (on average, the lowest compared to the other groups). BMI calculation is increasingly considered an imperfect tool due to its limitations, and more advanced parameters that may indicate existing abnormalities are emphasized, among which are adipose tissue or visceral fat content [18,65].

Considering these factors, the lowest risk in predicting obesity is among VEGE, especially the VEGAN group; VEGAN had the lowest values of BFM and PBF, as well as VAT. According to the current literature [66], visceral adipose tissue content, otherwise known as metabolically active adipose tissue, is highly correlated with cardiovascular disease, cancer, and diabetes risk. Regarding the study [46] on a smaller number of subjects: vegetarian (*n* = 20) and non-vegetarian (*n* = 20), where adipose tissue content was also measured, those on a vegetarian diet showed a lower value, but the results did not show statistical significance, which is consistent with the results obtained in the current study. PESCA and OMN show the highest results concerning BFM and VAT content. In the study by Jakše et al. [48], in addition to body weight and BMI, body fat [%] was compared, and all analyzed parameters were significantly higher in the non-vegans group (*n* = 29) than in the vegans group (*n* = 51). As with Vanacore et al. [55], the current study found the highest BFM scores among OMNs, while the lowest values relative to the parameter in question show differences. In the present study, the VEGAN group had the lowest values, while in Vanacore et al., vegetarians had the lowest values (vegans obtained intermediate values). The BFM content depends mainly on the quality of the diet and physical activity. Hence, the differences in the compared groups may result from more remarkable regularities for these two aspects in one of the groups.

In contrast, a small number of studies (Heiss et al. [51], Pinto et al. [56]) showed no differences concerning BMI and PBF values relative to omnivores and vegetarians (including vegans).

### 4.3. The Relation of the Behavioral Factors of the Study Groups to Anthropometric Analysis

#### 4.3.1. Muscle Content of the Studied Groups

The VEGAN group, despite the lowest physical activity relative to the other subjects, showed one of the highest values (next to the omnivore group) of muscle content (VEGAN 27.18 kg vs. OMN 27.96 kg, median 25.6 kg in both groups). It indicates an adequate amount of protein products in the diet, which is often considered a deficient component in plant-based diets. The vegan diet as a way of eating despite the lack of increased physical activity influenced the subjects to gain adequate muscle content. The study by Vanacore et al. [55] also analyzed muscle content among omnivores, vegetarians, and vegans, and the results differ significantly from the results of the current paper. Compared to vegans, omnivores scored substantially higher relative to muscle mass content. These differences could be explained as many people choosing a vegetarian/vegan diet do not know how to compose them properly. The VEGAN group of this present study confirmed adherence to a healthy diet in responses regarding the frequency of consumption (especially protein products, since these products mainly affect the building of muscle mass).

#### 4.3.2. Minerals Content of the Studied Groups

In a large cohort study by Tong et al. [54] including the white population of the United Kingdom (of each characteristic in 6 diet groups (198,166 regular meat eaters, 199,784 low meat eaters, 4381 poultry eaters, 9674 fish eaters, 6366 vegetarians, and 378 vegans) and British Indian population (in 2 diet groups (3322 meat eaters and 1186 vegetarians) showed significantly lower bone mineral density scores (also lower body fat in women) and blood pressure values in subjects designated as “non-red meat eaters”. In the white population, an additional result was shown in all five diet groups relative to “regular meat-eaters” with lower body weight, BMI, waist and hip circumferences, and body fat content.

Minerals content reflects the content of these components primarily in teeth and bones. Normal levels of minerals reduce the risk of osteoporosis or rickets [67]. The OMN and VEGAN groups showed this component’s highest average levels (3.48 kg OMN vs. 3.41 kg VEGAN). The Xie et al. study [62] measured Bone Mineral Density (BMD) using the CM-200 bone densitometer. The results also showed no differences between the study groups of vegans (*n* = 62), lacto-ovo vegetarians (*n* = 184), and omnivores (*n* = 246). Comparing the obtained highest levels of mineral content to the effects of the frequency of consumption, one can see some correlations that may affect the described outcome. VEGAN shows the highest intake of products that are sources of plant protein (tofu/tempeh, legumes, nuts, and seeds) and also provides with their dietary consumption sources of calcium (in addition to protein and iron) [68]. Despite the lack of its traditional sources, mainly dairy products such as cheese (their intake to the greatest extent in OMN group may account for the highest mineral content score). The study’s results [67] suggest a higher risk of fractures among vegans and vegetarians compared to those on a mixed diet, emphasizing the need for adequate intake of the described dietary components [69,70,71,72]. The studied group of vegans seems to meet these recommendations, which is not observed in the studied lacto/ovo-vegetarians.

#### 4.3.3. Comparison of Frequency of Consumption

The Barnard et al. study [73] showed a higher intake of fruits and vegetables and a lower intake of fat-laden or industrial foods than omnivores. Dorard and Matheu [64], following their cross-sectional study, conclude that vegetarian diets show an association with health benefits and are less concerned about problems related to body shape perception, including weight (mainly women). In the present study, the VEGAN group showed the correct dietary pattern through the frequency of consumption. Also, a statistical comparison of body composition parameters between groups showed significant differences, specifically between the VEGAN and VEGE groups, showing higher values of minerals and muscle tissue (SMM) among VEGANs. No statistical difference in the parameters of the body composition was found between different genders, but the obtained results showed that men in every group had higher values of muscle mass and also lower values of %fat which are which is in line with the expectations of gender differences [74].

Typically, vegans are perceived as a more restrictive subgroup of vegetarians. At the same time, many studies show significant differences between these groups in attitudes and behavioral or neurological factors. The eating habits of vegans are an essential component of the identity of this group. They show higher motivation to follow accepted dietary patterns (moral, personal, and pro-social motivation); the results of the present study confirm such observations [73,74,75,76].

Comparing the results of this work to the already mentioned results of Barnard et al. [73], the OMN group showed the most abnormalities concerning the food groups consumed in relation to the other groups. Similar results were also obtained in the study, as mentioned earlier by Rosi et al. [24], where vegans relative to vegetarians and omnivores consumed products (i.e., fruits, vegetables, legumes, and vegetable-based oils more frequently), while omnivores consumed alcoholic beverages or sweets and desserts most frequently relative to the other groups.

The PESCA and VEGE groups, in terms of frequency of food group intake, show a dietary pattern that diverges from the recommendations for healthy eating, with more inappropriate eating behaviors shown in these groups than those consistent with current recommendations. [7,60,61,77,78,79].

By dividing the study groups into subgroups of high (H) and low (L) frequency of consumption, additional calculations were also made to show the relationships between body composition parameters. The results obtained are as expected, except for the results about wine consumption in the PESCA and VEGAN groups. Consumption of this beverage in the “H” subgroup in VEGAN showed a correlation with lower muscle mass, which can be explained by the fact that wine is not a product that is a source of protein. Still, in PESCA, the “H” subgroup against this parameter showed increased values. It can be presumed that the resulting correlation is due to the fact that increased wine consumption in PESCA (subgroup “H”) also increased BMI and therefore resulted in increased body weight (perhaps, including muscle. Wine consumption in the PESCA group positively affected body composition parameters, while the opposite effect was seen in VEGANs. Wine, mainly red wine, contains some amounts of resveratrol, which may show beneficial health effects: antioxidant, cardioprotective, anti-inflammatory, and anti-cancer, as well as preventive against neurodegenerative diseases and obesity [80]. Some animal studies [81,82] show a positive effect on bone density or an increase in osteoblast activity, and it is possible that such a relationship also influenced the high mineral content in the PESCA group. It is also necessary to mention that wine is also a source of alcohol, excessive consumption of which leads to many disturbances, including death [83,84]. There is much controversy over the recommendations for wine consumption, and the need for further research on this issue is emphasized. In VEGANs, wine consumption showed the opposite effect, perhaps due to its lower consumption—the pescatarian group showed the highest average consumption of this product, or perhaps since resveratrol is also contained in products other than wine. The primary sources mentioned are red grapes, berries, and nuts [85], and the consumption (average) of the mentioned products was the highest among vegans.

The results of the Clarys et al. [34] study, which compared the overall diet quality of vegans, vegetarians, semi-vegetarians, pescatarians, and omnivores using the FFQ questionnaire, utilized the Healthy Eating Index (HEI-2010) and the Mediterranean Diet SCORE (MDS) as indicators for diet quality. Those on a vegan diet scored the highest, while those on a mixed diet scored the lowest. The results of a meta-analysis [27] of different studies (including a survey by Clarys et al.) comparing diet quality across dietary groups using the HEI-2010 show that 9 out of 12 studies indicate that vegetarian and vegan diets are of higher quality (scored higher) relative to omnivore diets [24,28,29,30,31,32,33,34,85,86,87,88,89].

The results of the current study confirm observations that people on vegan diets are not only characterized by a unique dietary style, but their behavioral patterns go beyond the assumptions of a healthy diet. Their goal is not only the absence of meat consumption but also behaviors beneficial to health and the environment with a greater good in mind. Diet is not an end for this group; it is a means to a higher end. Researchers even propose a description of this group as a social identity [63,64].

### 4.4. Strengths and Limitations

To the best of our knowledge, this is the first study comparing diet quality (assessment of eating habits) and body composition parameters among vegans, lacto/ovo-vegetarians, pescatarians, and traditional dieters. The lack of previous similar analyzes may be due to recruitment difficulties in these unique groups, as vegetarian diets and their variations are not standard dietary regimens. The beginning of veganism was noted in 1944 when The Vegan Society was established, so it is the dietary regimen approved in this century [90,91]. Recently, these diets have become better known and recognized by global institutions and organizations. The focus on these topics may be more significant in the world of science and show more health-promoting evidence.

- For the first time, this provides the opportunity to compare body composition between groups with entirely different dietary patterns and whether meat exclusion makes a difference. Some people may find the results surprising—the VEGAN group showed the level of SMM and minerals content almost as high as in the OMN group (the number of women and men in these groups was similar). There is a widespread concern that people who give up eating proteins of animal origin may have numerous deficiencies affecting the content of muscle mass or bone density. The obtained observations are highly promising. Therefore, there is a need to expand such research.
- Such a comparison can significantly enrich the knowledge of doctors, nutritionists, physiotherapists, and other specialists working with patients, especially regarding the perception of people on a vegan diet. The results obtained in this study enrich the current positive reports on the vegan diet, showing a high level of SMM and minerals content and the lowest values of body fat [kg, %], including VAT, which proves the impact of a well-balanced vegan diet for proper body shaping and contradicts the fear of perceiving this diet as deficient.
- The results of this study confirm the beneficial consequences of using a well-balanced vegan diet and maybe another argument “in favor” of pointing this diet as recommended. Specialists in various fields of medicine do not have to worry about the negative consequences of people on such (well-balanced) diets, but even that may consider recommending their use in some instances—especially when there is an excess of adipose tissue
- The study was conducted during a pandemic. However, the number of respondents compared to other studies is relatively high, considering how unique groups were recruited.
- The validated FFQ questionnaire allows the groups to be assessed concerning the presented eating behavior. Possible modifications were taken into account, e.g., adding plant substitutes for animal products to check the frequency of consumption in each study group (e.g., tofu, tempeh, vegetable yoghurt, egg substitutes), which was necessary due to the avoidance of traditional representatives of a given food group by some of the studied groups so that the assessment between the studied groups was as reliable as possible.
- Both the surveys and the body composition analysis were carried out in real life, not online.
- The modifications of the FFQ questionnaire, as well as the measurement of body composition, were performed by a qualified nutritionist.

This study also had some limitations:

- The main one was the lack of the use of additional tools, such as an intake diary (at least 24 h). Then, it would be possible to evaluate the diet quality more comprehensively, including the compliance calculation with the demand for essential macronutrients.
- The number of women and men in the study groups is random; the respondents are mainly women.
- No validated survey was used to assess behavioral factors, only a proprietary survey.
- It is necessary to mention that the result obtained from the analyzer (BIA method) may be influenced by, for example, the state of hydration, despite detailed recommendations issued before the examination.
- Possible mistakes may have resulted from self-defining the nutritional pattern
- The body composition analysis and the assessment of eating habits were performed once. This is a single sample study due to the timing of the pandemic.

## 5. Conclusions

**Scheme 2:**
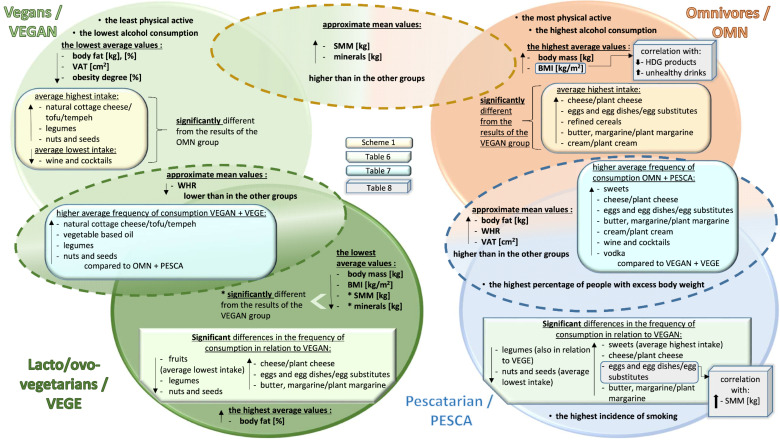
Summary of results of the study groups.

The findings indicated that the VEGAN group showed the most regularities regarding the observed behavioral factors and the frequency of consumption of selected food products. This was also reflected in the revealed body composition assessments, which is not an obvious issue. The omnivores group showed several positive behaviors, including the highest physical activity and the highest values on average (along with the VEGAN group) of SMM and minerals. However, in the overall behavioral factors assessment, most abnormalities were observed in this group compared to the recommendation. It is reflected in the body composition analysis results since the OMN group showed the highest mean results: body mass, BMI, and, together with the PESCA group (similar values): BFM, WHR, and VAT. VEGE and PESCA group in terms of the frequency of consumption do not necessarily comply with the current recommendations; additionally, VEGE differ significantly from the nutritional behavior of the VEGAN group (e.g., lower frequency of consumption of fruit, nuts, seeds, and legumes). The PESCA group also showed significant differences compared to the VEGAN group in the increased frequency of consumption, including sweets, cheese, butter/margarine, and the reduced frequency of consumption of legumes, nuts, and seeds. The PESCA group additionally declared the highest frequency of smoking cigarettes. Body composition analysis showed normal values, but some mean results compared to other groups were not very satisfactory. The summary of the results of this study is shown in Scheme 2. This study expands the knowledge about vegan diets’ health properties. It shows that this diet can enable one to maintain the correct values of body composition, including minerals and muscle content (our research indicates that these values of the VEGAN group are very similar to the OMN group and which are the highest compared to the other groups), This group also shows the benefits in terms of the lowest values of adipose tissue (BFM, PBF), including visceral fat (VAT), the excess of which is associated with a higher risk of cardiovascular diseases, cancer or diabetes. Besides, it can be presumed that diet had the most significant impact on this, as physical activity in the VEGAN group was the lowest compared to the rest of the respondents. The future direction of this work may encourage more in-depth comparing analyses between study groups, including more subjects (with a similar number of women and men), using additional research tools such as food diaries, and focusing on more in-depth questions about diet. It is also worth looking at the differences between vegans and lacto/ovo-vegetarians and underlining that not everyone following these diets is characterized by proper eating behavior.

## Figures and Tables

**Table 1:** Mortality for various diseases due to behavioral factors.

Causes	% Risk of Death for the Polish Population Due to Behavioral Factors	The Differences between the Results from Poland and the World
Total all causes	43.54	+5.30 p.p.
Cardiovascular diseases	52.36	+3.01 p.p.
Neoplasms	41.75	+5.05 p.p.
Chronic respiratory diseases	56.65	+11.36 p.p.

Own study based on Global Health Data Exchange (Institute for Health Metrics and Evaluation, GBD2019 (https://vizhub.healthdata.org/gbd-compare/, accessed on 5 August 2022) [6].

**Table 2:** Types of vegetarian diets with their characteristics and eliminated products.

Diet Type	Included Products				
	Plant Products (e.g., Grains, Fruits, Vegetables, Legumes)	Dairy Products	Eggs	Fish and Seafood	Meat (Occasional, Mainly Poultry)
Semivegetarian	Yes	Yes	Yes	Yes	Yes
Pescatarian	Yes	Yes	Yes	Yes	No
Vegetarian	Yes	Yes, with exceptions (lacto-vegetarian or lacto-ovo-vegetarian-yes, ovo-vegetarian- no)	Yes, with exceptions (ovo-vegetarian or lacto-ovo-vegetarian variety-yes, lacto-vegetarian- no)	No	No
Vegan	Yes	No	No	No	No
Raw vegan	Yes, only raw	No	No	No	No
Fruitarian	Yes, only raw fruits	No	No	No	No

Sources: Own study based on: Melina, V.; Craig, W.; Levin, S. Position of the Academy of Nutrition and Dietetics: Vegetarian Diets. Journal of the Academy of Nutrition and Dietetics 2016, 116, 1970–1980 [19].

**Table 3:** Baseline characteristics of the participants.

Variables	Studied Groups				p *
	VEGAN	VEGE	PESCA	OMN	
	*n* = 53 F/M: 37/17 % F: 67.9	*n* = 52 F/M: 48/4 % F: 92.3	*n* = 28 F/M: 24/4 % F: 85.7	*n* = 43 F/M: 27/16 % F: 62.8	
**Age, years**	**mean ± SD**				0.120
	30.2 ± 8.3	28.1 ± 7.6	31.0 ± 10.1	30.0 ± 6.1	
**Exercise, level**	*n* (%) **				0.377
low activity [<150 min/week]	11 (20.7)	7 (13.5)	2 (7.1)	5 (11.7)	
medium activity [150–300 min weekly]	32 (60.4)	36 (69.2)	21 (75.0)	25 (58.1)	
high activity [>300 min/week]	10 (18.9)	9 (17.3)	5 (17.9)	13 (30.2)	
**Smoking**	*n* (%) **				0.060
never	44 (83.0)	40 (76.9)	16 (57.1)	36 (83.7)	
Occasionally [approx. 1–3 cigarettes/week]	4 (7.5)	8 (15.4)	7 (25.0)	1 (2.3)	
regularly small amounts [less than a pack/day]	3 (5.7)	4 (7.7)	5 (17.9)	6 (14.0)	
regularly large amounts [more than a pack/day]	2 (3.8)	0 (0.0)	0 (0.0)	0 (0.0)	
**Sleep, h/night**	*n* (%) **				0.821
≤6	6 (11.3)	13 (25.0)	4 (14.3)	7 (16.3)	
6–8	39 (73.6)	35 (67.3)	23 (82.1)	33 (76.7)	
≥9	8 (15.1)	4 (7.7)	1 (3.6)	3 (7.0)	
**Vodka consumption**	*n* (%) **				0.116
never	40 (75.5)	34 (65.4)	18 (64.3)	22 (51.2)	
rare	10 (18.9)	13 (25.0)	5 (17.8)	15 (34.9)	
monthly	3 (5.6)	5 (9.6)	4 (14.3)	6 (13.9)	
weekly	0 (0.0)	0 (0.0)	1 (3.6)	0 (0.0)	
daily	0 (0.0)	0 (0.0)	0 (0.0)	0 (0.0)	

F/M—female/male. * *p* was calculated to evaluate the differences between groups concerning selected variables. ** *n* (%)-the number of participants and their percentage content in the study group.

**Table 4:** Characteristics of the main body composition parameters in the studied groups.

Body Component	Studied Groups				p *
	VEGAN	VEGE	PESCA	OMN	
Minerals [kg]	3.41 ± 0.61 F: 3.10 ± 0.33 M: 4.08 ± 0.55	3.14 ± 0.42 F: 3.07 ± 0.33 M: 4.03 ± 0.38	3.26 ± 0.52 F: 3.12 ± 0.39 M: 4.12 ± 0.33	3.48 ± 0.70 F: 3.09 ± 0.44 M: 4.13 ± 0.56	0.048 F: 0.734 M: 0.932
BFM [kg]	13.60 ± 4.77 F: 14.3 ± 4.49 M: 12.1 ± 5.11	14.6 ± 3.53 F: 14.7 ± 3.55 M: 13.6 ± 3.61	15.02 ± 5.18 F: 14.8 ± 5.37 M: 16.2 ± 4.23	15.03 ± 4.93 F: 15.0 ± 4.31 M: 15.1 ± 5.99	0.391 F: 0.926 M: 0.263
Body mass [kg]	62.75 ± 10.3 F: 58.1 ± 6.33 M: 72.6 ± 10.4	58.84 ± 7.47 F: 57.8 ± 6.49 M: 71.7 ± 7.04	61.15 ± 11.0 F: 58.5 ± 9.30 M: 76.8 ± 6.62	65.32 ± 13.7 F: 58.5 ± 9.19 M: 76.8 ± 12.4	0.112 F: 0.932 M: 0.586
BMI [kg/m^2^]	21.16 ± 2.4 F: 20.5 ± 2.10 M: 22.6 ± 2.42	20.94 ± 1.97 F: 20.9 ± 2.01 M: 21.8 ± 1.42	21.28 ± 2.53 F: 21.1 ± 2.58 M: 23.1 ± 1.19	22.00 ± 3.00 F: 20.9 ± 2.21 M: 23.8 ± 3.32	0.512 F: 0.800 M: 0.516
PBF [%]	21.76 ± 7.01 F: 24.4 ± 6.11 M: 16.2 ± 5.50	24.82 ± 4.73 F: 25.3 ± 4.48 M: 18.8 ±. 3.53	24.12 ± 5.98 F: 24.6 ± 6.16 M: 21.0 ± 3.99	23.02 ± 5.87 F: 25.3 ± 4.64 M: 19.1 ± 5.76	0.077 F: 0.845 M: 0.242
SMM [kg]	27.18 ± 5.83 F: 23.9 ± 2.64 M: 34.1 ± 4.46	24.16 ± 3.68 F: 23 5 ± 2.79 M: 32.6 ± 2.47	25.33 ± 4.74 F: 23 8 ± 3.06 M: 34.3 ± 2.58	27.96 ± 6.83 F: 23.8 ± 3.66 M: 35.0 ± 4.89	0.019 F: 0.573 M: 0.818
WHR	0.83 ± 0.04 F: 0.82 ± 0.04 M: 0.86 ± 0.05	0.83 ± 0.04 F: 0.82 ± 0.04 M: 0.84 ± 0.03	0.84 ± 0.044 F: 0.83 ± 0.04 M: 0.89 ± 0.01	0.85 ± 0.046 F: 0.83 ± 0.03 M: 0.88 ± 0.04	0.177 F: 0.797 M: 0.104
VAT [cm^2^]	5.42 ± 2.41 F: 5.75 ± 2.29 M: 4.71 ± 2.59	5.67 ± 1.71 F: 5.73 ± 1.7 M: 5.00 ± 1.83	5.89 ± 2.25 F: 5.75 ± 2.33 M: 6.75 ± 1.71	5.86 ± 2.43 F: 5.85 ± 2.11 M: 5.88 ± 2.96	0.670 F: 0.975 M: 0.368

The results are presented as mean ± SD. * *p* was calculated to evaluate the differences between groups by considering all participants, regardless of gender, and then analyzes were performed to check the possible differences in the groups within gender. Abbreviations: F—female, M—male; BFM—Body Fat Mass; BMI—Body Mass Index; PBF—Percentage Body Fat; SMM—Skeletal Muscle Mass; WHR—Waist-Hip Ratio; VAT—Visceral Adipose Tissue.

**Table 5:** Characteristics of the study participants concerning the BMI classification.

Characteristics	VEGAN		VEGE		PESCA		OMN	
	*n*	%	*n*	%	*n*	%	*n*	%
Underweight (<18.5 kg/m^2^)	7	13.21	6	11.54	3	10	4	9.30
Normal weight (≥18.5 kg/m^2^ to <25.0 kg/m^2^)	41	77.36	46	88.46	23	76.67	33	76.74
Overweight (≥25.0 to <30.0 kg/m^2^)	5	9.43	0	-	4	13.33	6	13.95

**Table 6:** Statistically significant differences in the frequency of consumption of food products between the studied groups.

Food Products	Pairwise Comparisons	*p*
Sweets	PESCA > VEGAN	0.029
Natural cottage cheese/tofu/tempeh	OMN < VEGAN OMN < VEGE	<0.001 <0.001
Cheese (hard cheese, blue cheese, processed cheese)/plant cheese	OMN > VEGAN PESCA > VEGAN VEGE > VEGAN	<0.001 0.001 <0.001
Eggs and egg dishes/egg substitute	OMN > VEGAN PESCA > VEGAN VEGE > VEGAN	<0.001 <0.001 <0.001
Refined cereals (white bread, rye, wheat-rye bread, toast bread, white bread rolls, brioche, bagels)	OMN > VEGAN	0.043
Butter, margarine/plant-based margarine	OMN > VEGAN PESCA > VEGAN VEGE > VEGAN	<0.001 0.012 <0.001
Cream (single, double, sour)/plant-based cream	OMN > VEGAN	0.013
Fruits (raw)	VEGE < VEGAN	0.041
Legumes (green peas, beans, lentils)	OMN < VEGAN PESCA < VEGAN PESCA < VEGE VEGE < VEGAN	<0.001 <0.001 <0.001 0.006
Nuts and seeds	OMN < VEGAN PESCA < VEGAN VEGE < VEGAN	<0.001 0.002 0.023
Wine and cocktails	OMN > VEGAN	0.039

**Table 7:** Statistically significant differences in the frequency of consumption between the studied groups divided into two categories—OMN + PESCA (including meat consumption) and VEGE + VEGAN (not including meat consumption).

Frequency of Consumption with Significant Differences between Groups Mean (Median)			
OMN + PESCA > VEGE + VEGAN	*p*	VEGE + VEGAN > OMN + PESCA	*p*
**Sweets** 3.58 (4.00) > 3.26 (3.00)	0.024	**Natural cottage cheese/tofu/tempeh** 3.70 (4.00) > 3.14 (3.00)	<0.001
**Cheese (hard cheese, blue cheese, processed cheese)/plant-based cheese** 3.46 (4.00) > 2.74 (3.00)	<0.001	**Vegetable-based oil (olive oil, rapeseed oil)** 4.46 (5.00) > 4.23 (4.00)	0.036
**Eggs and egg dishes/egg substitutes** 3.59 (4.00) > 2.15 (3.00)	<0.001	**Legumes (green peas, beans, lentils)** 4.03 (4.00) > 3.25 (3.00)	<0.001
**Butter, margarine/plant-based** **margarine** 3.10 (3.00) > 2.23 (2.00)	<0.001	**Nuts and seeds** 4.09 (4.00) > 3.51 (4.00)	<0.001
**Cream (single, double, sour)/plant-based cream** 2.30 (2.00) > 1.98 (2.00)	0.018		
**Wine and drinks** 2.45 (3.00) > 2.17 (2.00)	0.028		
**Vodka** 1.61 (1.00) > 1.37 (1.00)	0.039		

**Table 8:** Correlations between the body composition parameters and the product consumption frequency.

Groups	Product	Body Composition Parameter	Spearman’s *ρ*	*p*
**OMN**	Sweets	Minerals [kg] SMM [kg]	−0.332 −0.316	0.030 0.039
	HDG products *	BMI [kg/m^2^]	−0.321	0.036
	Unhealthy drinks **	BMI [kg/m^2^] VAT [cm^2^]	0.312 0.351	0.041 0.021
**PESCA**	Eggs and egg dishes/egg substitute	SMM [kg]	0.410	0.030
**VEGE**	Sugar	BMI [kg/m^2^] WHR	0.278 0.328	0.048 0.019
	high in sugar products ***	BMI [kg/m^2^]	0.362	0.009
**VEGAN**	Sweets	Minerals [kg] SMM [kg]	−0.323 −0.341	0.018 0.012
	Instant soups/meals	Minerals [kg] SMM [kg]	−0.290 −0.285	0.035 0.038

Abbreviations: SMM—Skeletal Muscle Mass; HDG (Healthy Diet Guidelines); BMI—Body Mass Index; VAT—Visceral Adipose Tissue; WHR—Waist-Hip Ratio. * HDG (Healthy Diet Guidelines) products [60,61], including plant protein products: fruits (raw), processed fruits, vegetables, legumes, wholemeal cereals, refined cereals, milk and natural milk beverages/natural plant beverages, natural cottage cheese/tofu/tempeh, natural yoghurt/natural plant yoghurt, nuts and seeds. ** unhealthy drinks: beer, wine, cocktails, vodka, energy drinks. *** high in sugar products: sugar, sweets, fruit juices and nectars, sweetened milk beverages/sweetened plant yoghurts.

**Table 9:** Relationships between the parameters of body composition analysis and the high and low frequency of food consumption in the studied groups. The data included only statistically significant results.

Product	Body Composition Parameter	*p*
**OMN group**		
Milk and natural milk beverages (kefir, buttermilk)/natural plant-based beverages	**Body mass [kg]:** H 63.13 vs. L 70.36 [median: H 60.00 vs. L 66.40]	0.040
Wholemeal cereals (wholemeal wheat or rye bread, seeded loafs, pumpernickel)	**WHR:** H 0.84 vs. L 0.87 [median: H 0.83 vs. L 0.86]	0.050
Coarse groats (buckwheat groats, barley), brown rice, wholemeal pasta	**Body mass [kg]:** H 60.44 vs. L 68.51 [median: H 59.10 vs. L 68.10]	0.043
Nuts and seeds	**BMI [kg/m^2^]:** H 21.13 vs. L 22.99 [median: H 20.20 vs. L 23.75] **PBF [%]:** H 24.41 vs. L 21.41 [median: H 24.30 vs. L 21.35] **WHR:** H 0.83 vs. L 0.86 [median: H 0.83 vs. L 0.85]	0.028 0.047 0.035
Beer	**WHR:** H 0.89 vs. L 0.84 [median: H 0.89 vs. L 0.83]	0.032
**PESCA group**		
Sugar	**Body mass [kg]:** H 66.54 vs. L 59.35 [median: H 66.70 vs. L 59.30] **WHR:** H 0.86 vs. L 0.83 [median: H 0.87 vs. L 0.84]	0.050 0.046
Sugar substitute (xylitol, erythritol, etc.)	**BFM [kg]:** H 11.93 vs. L 15.85 [median: H 10.30 vs. L 15.70] **VAT [cm^2^]:** H 4.33 vs. L 6.31 [median: H 4.00 vs. L 6.50]	0.034 0.027
Fish	**Minerals [kg]:** H 3.55 vs. L 3.19 [median: H 3.45 vs. L 3.12]	0.049
Wine and cocktails	**Minerals [kg]:** H 3.80 vs. L 3.19 [median: H 3.62 vs. L 3.12] **BMI [kg/m^2^]:** H 24.13 vs. L 20.93 [median: H 23.60 vs. L 21.20] **SMM [kg]:** H 30.00 vs. L 24.77 [median: H 29.10 vs. L 23.40]	0.024 0.024 0.043
**VEGE group**		
Sugar	**Body mass [kg]:** H 61.39 vs. L 57.31 [median: H 62.40 vs. L 57.20]	0.028
Natural cottage cheese/tofu/tempeh	**SMM [kg]:** H 24.78 vs. L 23.05 [median: H 23.60 vs. L 22.40]	0.050
Sweetened milk beverages/sweetened plant-based yoghurts	**BMI [kg/m^2^]:** H 22.36 vs. L 20.76 [median: H 21.60 vs. L 20.70] **PBF [%]:** H 28.82 vs. L 24.33 [median: H 26.60 vs. L 24.45]	0.039 0.039
Wholemeal cereals (wholemeal wheat or rye bread, seeded loafs, pumpernickel)	**BMI [kg/m^2^]:** H 20.73 vs. L 21.60 [median: H 20.55 vs. L 21.60]	0.041
**VEGAN group**		
Sweets	**Minerals [kg]:** H 3.21 vs. L 3.52 [median: H 3.16 vs. L 3.38] **SMM [kg]:** H 25.35 vs. L 28.20 [median: H 24.30 vs. L 27.30]	0.050 0.039
Milk and natural milk beverages (kefir, buttermilk)/natural plant-based beverages	**BFM [kg]:** H 14.09 vs. L 11.18 [median: H 13.45 vs. L 9.60]	0.036
Breakfast cereals (muesli, cornflakes, oat flakes)	**BFM [kg]:** H 11.97 vs. L 14.59 [median: H 11.45 vs. L 14.30] **Body mass [kg]:** H 59.99 vs. L 64.43 [median: H 58.70 vs. L 63.00] **VAT [cm^2^]:** H 4.65 vs. L 5.87 [median: H 4.50 vs. L 5.00]	0.024 0.049 0.043
Processed fruit (dried, boiled)	**PBF [%]:** H 19.87 vs. L 23.32 [median: H 20.50 vs. L 23.30]	0.037
Nuts and seeds	**BFM [kg]:** H 12.94 vs. L 16.42 [median: H 12.80 vs. L 17.45] **PBF [%]:** H 20.87 vs. L 25.57 [median: H 21.50 vs. L 26.05]	0.026 0.033
Wine and cocktails	**Minerals [kg]:** H 2.88 vs. L 3.44 [median: H 3.06 vs. L 3.31] **SMM [kg]:** H 21.70 vs. L 27.51 [median: H 23.70 vs. L 25.80]	0.047 0.036
Instant soups/meals	**Minerals [kg]:** H 2.70 vs. L 3.44 [median: H 2.70 vs. L 3.31] **SMM [kg]:** H 20.20 vs. L 27.45 [median: H 20.20 vs. L 25.80]	0.028 0.026

Abbreviations: WHR—Waist-Hip Ratio; SMM—Skeletal Muscle Mass; HDG—Healthy Diet Guidelines; BMI—Body Mass Index; VAT—Visceral Adipose Tissue.

## Data Availability

All necessary data are included in the paper.

## Supplementary Materials

The following supporting information can be downloaded at: https://www.mdpi.com/article/10.3390/nu14214591/s1. Questionnaire S1: Self-questionnaire: Diet and behavioral factors questionnaire; Questionnaire S2: Modified questionnaire FFQ-6; Questionnaire S3: A Delphi interview; Table S1: Detailed data on the responses to the questionnaires of the analyzed groups.
