# Supplementary material for: Nutritional Status and Habits among People on Vegan, Lacto/Ovo-Vegetarian, Pescatarian and Traditional Diets
Source: Nutrients. 2022 Nov 1;14(21):4591. doi: 10.3390/nu14214591 (PMC9657343; doi:10.3390/nu14214591)
Supplement: Supplementary file 1 [file nutrients-14-04591-s001.zip › suppl 1.pdf]

Questionnaire S1: Self-questionnaire: Diet and behavioral factors questionnaire

Patient number.............

Year of birth::……………………

Gender: female/male

Diet followed:

A. veganism (plant diet)

B. vegetarianism (lacto-ovo) *Duration of veganism/vegetarianism ……[years]

C. pescatarianism

D. mixed/traditional

E other:……………………………………

Physical activity: LITTLE / MODERATE / HIGH

Sleep duration: …………………………… hours

Diseases or ailments:

…………………………………………………………………………………………………….

…………………………………………………………………………………………………….

Medications / dietary supplements taken:

……………………………………………………………………………………………………

…………………………………………………………………………………………………….
