# Supplementary material for: Nutritional Status and Habits among People on Vegan, Lacto/Ovo-Vegetarian, Pescatarian and Traditional Diets
Source: Nutrients. 2022 Nov 1;14(21):4591. doi: 10.3390/nu14214591 (PMC9657343; doi:10.3390/nu14214591)
Supplement: Supplementary file 1 [file nutrients-14-04591-s001.zip › suppl 2.pdf]

Questionnaire S2: Modified questionnaire FFQ-6

Consumption frequency. In the past 12 months, how often did you eat or drink:

**Products**	**Never or almost never**	**Once a month or less**	**Several times a month**	**Several times a week**	**Daily**
Codes of consumption frequency:	(1)	(2)	(3)	(4)	(5)
Sugar					
Sugar substitute (xylitol, erythritol, etc.)					
Sweets					
Savoury snacks					
Milk and natural milk beverages (kefir, buttermilk)/natural plant-based beverages					
Natural cottage cheese/ tofu / tempeh					
Natural yoghurt/natural plant-based yoghurt					
Sweetened milk beverages/sweetened plant yoghurts					
Cheese (hard cheese, blue cheese, processed cheese)/ plant-based cheese					
Eggs and egg dishes/ egg substitutes					
Wholemeal cereals (wholemeal wheat or rye bread, seeded loafs, pumpernickel)					
Refined cereals (white bread, rye, wheat-rye bread, toast bread, white bread rolls, brioche, bagels)					
Coarse groats (buckwheat groats, barley), brown rice, wholemeal pasta					
Fine groats (semolina, milled barley), white rice, pasta					
Breakfast cereals (muesli, cornflakes, oat flakes)					
Vegetable based oil (olive oil, rapeseed oil)					
Butter, margarine/ plant-based margarine					
Cream (single, double, sour)/ plant-based cream					
Mayonnaise and salad dressings/ plant-based dressings					
Fruits (raw)					
Processed fruit (dried, boiled)					
Vegetables (raw)					
Legumes (green peas, beans, lentils)					
Nuts and seeds					
**Poultry*					
**Red meat*					

**Cured meats (ham, cold meat)*					
**Sausages*					
**Fish*					
Fruit juices and nectars					
Vegetable juices					
Beer					
Wine and cocktails					
Vodka					
Energy drinks					
"Fast food" food					
Instant soups/meals eg. amino/knor meals					

*- QUESTION ONLY FOR PEOPLE ON A OMNIVOROUS/TRADITIONAL DIET
