# Supplementary material for: Nutritional Status and Habits among People on Vegan, Lacto/Ovo-Vegetarian, Pescatarian and Traditional Diets
Source: Nutrients. 2022 Nov 1;14(21):4591. doi: 10.3390/nu14214591 (PMC9657343; doi:10.3390/nu14214591)
Supplement: Supplementary file 1 [file nutrients-14-04591-s001.zip › suppl 3.pdf]

Questionnaire S3: A Delphi interview

1) Is it worthwhile to perform body composition analysis in patients?
   a. Yes, definitely
   b. Yes, but the measurement of body weight and circumference itself is equally precise
   c. No

2) Do you think that the body composition analysis using the BIA (electrical bioimpedance) method is a better tool to assess the nutritional status than possible anthropometric parameters, including body circumference measurements, body weight, height, and fold meter tests?
   a. Yes
   b. No
   c. Equally reliable
   d. I don't know

3) What do you value the most from the body composition analyzer?
   a. Precise/accurate result
   b. Speed of measurement
   c. Numerous parameters displayed
   d. All of the above answers are correct
   e. I do not think this result is a good way to assess nutritional status

4) Does the assessment of eating habits using the frequency of consumption questionnaire (FFQ) seem correct?
   a. Yes
   b. No
   c. I don't know the FFQ

5) What do you consider to be a better method to assess eating habits / diet?
   a. Food diary
   b. FFQ questionnaire
   c. (based on) the patient's regular diary
   d. Other ....

6) What do you consider to be a better method to assess eating habits/diet <u>if the group of respondents is assessed? (group evaluation, not individual evaluation)</u>?
   a. Food diary
   b. FFQ questionnaire
   c. (based on) the patient's regular diary
   d. Other ....

7) Do you think that proper nutrition increases the content of muscles, the content of protein and minerals, and reduces body fat?
   a. Yes
   b. No
   c. I don't know

8) Do you think that people on vegetarian diets may show more correct results in terms of compliance with the principles of a healthy diet (checked using the FFQ form) and body composition analysis parameters?
   a. Yes

b. No
c. I don’t know

9) How do you think which of the surveyed groups: people on a traditional diet, pescatarian, vegetarians or vegans, will show the most regularities about their lifestyle?
a. People on a traditional diet
b. Pescatarian
c. Vegetarians
d. Vegans

10) How do you think which of the surveyed groups: people on a traditional diet, pesovegetarians, vegetarians or vegans, will show the most correct results of the parameters of the body composition analysis?
a. People on a traditional diet
b. Pescatarian
c. Vegetarians
d. Vegans

11) How do you think which of the surveyed groups: people on a traditional diet, pesovegetarians, vegetarians, or vegans, will show the highest values of body weight, BMI and WHR, and fat content, including visceral fat?
a. People on a traditional diet
b. Pescatarian
c. Vegetarians
d. Vegans

12) How do you think which of the surveyed groups: people on a traditional diet, pesovegetarians, vegetarians, or vegans, will show the lowest values of body weight, BMI and WHR, and fat content, including visceral fat?
a. People on a traditional diet
b. Pescatarian
c. Vegetarians
d. Vegans

13) How do you think which of the surveyed groups: people on a traditional diet, pesovegetarians, vegetarians, or vegans, will eat sweets most often?
a. People on a traditional diet
b. Pescatarian
c. Vegetarians
d. Vegans

14) Do you agree that consuming sweets may reduce the body composition parameters, i.e., the content of water, protein, minerals, and muscle mass?
a. Yes
b. No
c. I don’t know

15) How do you think which of the surveyed groups: people on a traditional diet, pesovegetarians, vegetarians or vegans, will most often consume products from the natural cottage cheese / tofu / tempeh category?
a. People on a traditional diet
b. Pescatarian
c. Vegetarians

d. Vegans

16) How do you think which of the surveyed groups: people on a traditional diet, pesovegetarians, vegetarians or vegans, will most often eat the following products: fruit, legumes, nuts and seeds?
    a. people on a traditional diet
    b. pescatarian
    c. vegetarians
    d. vegans
